# Advance Care Planning and Health-Related Quality of Life in Huntington Disease: Results from a Multicenter National Study

**DOI:** 10.1089/pmr.2022.0034

**Published:** 2023-03-22

**Authors:** Leonard L. Sokol, Jonathan P. Troost, Danny Bega, Benzi M. Kluger, Holly G. Prigerson, Martha Nance, Samuel Frank, Joel S. Perlmutter, Praveen Dayalu, David Cella, Noelle E. Carlozzi

**Affiliations:** ^1^ The Ken and Ruth Davee Department of Neurology, Northwestern University Feinberg School of Medicine, Chicago, Illinois, USA.; ^2^ McGaw Bioethics Scholars Program, Center for Bioethics and Humanities, Northwestern University Feinberg School of Medicine, Chicago, Illinois, USA.; ^3^ Division of Palliative Medicine, Department of Medicine, University of California, San Francisco, San Francisco, California, USA.; ^4^ Michigan Institute for Clinical and Health Research, University of Michigan, Ann Arbor, Michigan, USA.; ^5^ Department of Neurology, University of Rochester Medical Center, Rochester, New York, USA.; ^6^ Department of Medicine, University of Rochester Medical Center, Rochester, New York, USA.; ^7^ Cornell Center for Research on End-of-Life Care, Weill Cornell Medicine, New York, New York, USA.; ^8^ Struthers Parkinson's Center, Golden Valley, Minnesota, USA.; ^9^ Department of Neurology, Beth Israel Deaconess Medical Center, Harvard Medical School, Boston, Massachusetts, USA.; ^10^ Neurology, Radiology, Neuroscience, Physical Therapy and Occupational Therapy, Washington University in St. Louis, St. Louis, Missouri, USA.; ^11^ Department of Neurology, University of Michigan, Ann Arbor, Michigan, USA.; ^12^ Department of Medical Social Sciences, Northwestern University Feinberg School of Medicine, Chicago, Illinois, USA.; ^13^ Department of Physical Medicine and Rehabilitation, University of Michigan, Ann Arbor, Michigan, USA.

**Keywords:** advance care planning, end-of-life planning, Huntington disease, neuropalliative

## Abstract

**Objective:**

With Huntington disease (HD), a fatal neurodegenerative disease where the prevalence of suicidal thoughts and behavior (STB) remains elevated as compared to other neurological disorders, it is unknown whether STB and health-related quality of life (HRQoL) affect plans for the end of life or more broadly, advance care planning (ACP). Conversely, it is unknown whether ACP would provoke future changes to STB and HRQoL. Therefore, we sought to evaluate whether STB and HRQoL patient-reported outcomes (PROs) contribute to ACP and whether ACP relates to changes in STB and HRQoL at 24 months.

**Methods:**

HD-validated clinician- and patient-assessments (i.e., HRQoL PROs) were obtained at baseline enrollment, 12 and 24 months through our multi-center study (HDQLIFE™) throughout the United States among people with premanifest, early-stage, and late-stage manifest HD. We used linear mixed-effects models to determine the relationships between STB and HRQoL at baseline and HDQLIFE End of Life Planning at follow-up. Separate linear mixed-effects models were used to assess the relationship between HDQLIFE End of Life Planning at baseline, and HRQoL and STB at 12 and 24 months. False discovery rate adjustments were used to account for multiple comparisons.

**Results:**

At baseline enrollment, STB and HRQoL were not related to HDQLIFE End of Life Planning at 12 or 24 months. Similarly, at baseline, HDQLIFE End of Life Planning demonstrated no association with STB or HRQoL at 12 or 24 months.

**Interpretation:**

STB and HRQoL PROs do not significantly affect patient engagement with ACP. Most importantly, engaging in ACP does not cause untoward effects on HRQoL or STB for this rare neurodegenerative disease where the lifetime prevalence of STB approaches 30%.

## Introduction

Advance care planning (ACP) is defined as “a process that consists of many behaviors, such as choosing a surrogate decision-maker, defining values and preferences for medical care, and communicating those wishes to others.”^1^ In Huntington disease (HD), a hereditary and ultimately fatal neurodegenerative disease affecting 7 in 100,000 people worldwide,^2,3^ the literature on ACP is sparse.^4,5^ Our group previously determined that people with HD do not engage in ACP at a rate higher than the general adult population.^4^ We also identified that higher education, older age, and late-stage HD were associated with completing advance directives.^4^

Despite knowledge of these associated factors with advanced directive completion in HD, clinicians who care for people with HD and their families may nonetheless shy away from ACP discussions, believing that broaching discussion about the end of life may increase patient discomfort. However, among other serious illnesses, ACP discussion has not shown adverse psychosocial outcomes.^6^ Yet, whether those findings apply to HD remains unclear, as people with HD differ in their burden of psychiatric and existential symptoms and the capacity to cope, given progressive cognitive and psychiatric dysfunction.

Specifically, people with all stages of HD exhibit elevated rates of hopelessness,^7^ existential distress,^8^ and death anxiety.^9,10^ Further, HD has one of the highest rates of suicidal thoughts and behavior (STB) among all neurological disorders, and suicide is a major cause of death.^11^ Indeed, a comprehensive assessment surrounding the association among ACP, STB, and health-related quality of life (HRQoL) has been lacking in HD.

Data relating ACP with STB and HRQoL might help frame ACP discussions with stakeholders who are fearful of provoking adverse emotional outcomes by engaging in these discussions. Similar concerns pervaded the field of suicidology, where some practitioners falsely presumed that talking about suicide among those with suicidal thoughts might precipitate worsening future STB. Yet, research has not corroborated that hypothesis.^12^

Understanding how STB and HRQoL may affect the engagement of ACP and conversely, how engaging in ACP activities may affect future changes to STB and HRQoL within HD could guide clinician's practices through understanding actual risks of ACP for this vulnerable population. Such knowledge would be vital to clinical care, provider–patient communication, and long-term planning for patients, clinicians, and other family members.

We evaluate the following two objectives: whether baseline enrollment levels of STB and mental, physical, or social aspects of HRQoL were associated with ACP and how ACP engagement effects STB and HRQoL at 12- and 24-month follow-ups. We hypothesized that mental and social HRQoL would predict ACP engagement and that, consistent with past literature among chronic diseases,^13^ ACP would have modest improvements in mental and social HRQoL patient-reported outcomes (PROs) at 12 and 24 months without worsening STB.

## Methods

### Participants

HDQLIFE™ was an observational, longitudinal study dedicated to creating and validating HRQoL PROs specific to people with the HD genetic mutation (prodromal, *n* = 50; early-stage, *n* = 171, and late-stage manifest HD, *n* = 101). This study was conducted from 2012 to 2016 across several academic medical centers in diverse geographic regions within the United States. A portion of the HDQLIFE study sample was collected concurrently with the PREDICT-HD study, a study designed to identify the earliest clinical features of HD before phenoconersion.^14^

Eligibility criteria included those whose primary language was English, 18 years or older, or who had documented *HTT* gene mutation with ≥36 CAG repeats (additional inclusion/exclusion criteria may be found here^15^). Recruitment occurred through advertisements, the national HD roster obtained through Indiana University, skilled nursing facilities, and academic neurology clinics. Each study site received approval from its respective Institutional Review Board. All participants provided informed consent at baseline enrollment, excluding those with cognitive impairment unable to provide consent.

### Procedure

Participants completed clinician- and patient-rated assessments at baseline, 12 and 24 months (details surrounding the psychometric validation of the HD clinician-rated assessments and patient-rated Neuro-QoL/PROMIS^®^/HDQLIFE PROs may be found elsewhere^15–25^). PROs were administered as computer adaptive tests, when available, plus short forms (SFs), either in a clinical setting or remotely via the Internet (*Note.* HDQLIFE End of Life Planning and HDQLIFE Meaning and Purpose are only available as SFs).

#### Clinician-rated assessments

The HDQLIFE assessment battery included clinician-rated assessments at each visit. These involved HD-trained clinicians, who administered the Unified Huntington Disease Rating Scale (UHDRS^®^)^26^ and short Problem Behavior Assessment (PBA-s) forms.^27^

We examined components of the motor scale and the Total Functional Capacity (TFC) from the UHDRS. The “diagnostic confidence level” (DCL) item from the motor scale was examined. The DCL is a single clinician-rated item on a 5-point Likert scale (range from 0 to 4), representing the confidence that the motor signs reflect manifest HD. Ratings of 4 indicate a 98%–100% chance of manifesting HD, defined as “unequivocal.”

Values <4 on the DCL signify prodromal HD. We also examined the TFC from the UHDRS, which scored from 0 to 13 (higher scores indicate greater independence across a range of activities of daily living). The TFC was used to determine the HD stage for individuals with manifest HD (i.e., those with a DCL of 4). Early-stage manifest HD is defined as a TFC score ranging from 7 to 13, and a TFC score <7 indicates late-stage manifest HD.^28^

The PBA-s comprises 11 different behavioral/cognitive/psychiatric items (including an item on suicidality) within HD, rated by the clinician, including patient and collateral support (e.g., care partner) when available. Each question inquires about the (1) frequency and the (2) severity of the symptom/domain within the last three weeks. The frequency and severity are scored on a 5-point Likert scale, ranging from 0 (complete absence) to 4 (present every day). Each of the 11 items on the PBA-s is computed by multiplying the frequency by the severity. For our study, we examined scores on the PBA-suicide item.

### Patient-rated assessments

Our study categorized assessments into four HRQoL categories: physical, mental, social, and cognitive. The physical domain included five PROs: (1) HDQLIFE Chorea, (2) HDQLIFE Swallowing difficulties, (3) HDQLIFE Speech difficulties, (4) Neuro-QoL Upper Extremity Function, and (5) Neuro-QoL Lower Extremity Function. The mental HRQoL category included seven PROs and one clinician-rated assessment: (1) PROMIS Anger, (2) HDQLIFE Meaning and Purpose, (3) HDQLIFE Concern with Death and Dying, (4) Neuro-QoL Anxiety, (5) Neuro-QoL Depression, (6) Neuro-QoL Emotional and Behavioral Dyscontrol, (7) Neuro-QoL Positive Affect and Well-being, and (8) the clinician-rated (PBA-s) item on suicide (PBA-suicide). The social HRQoL domain included three PROs: (1) Neuro-QoL Ability to Participate in Social Roles and Activities; (2) Neuro-QoL Satisfaction with Social Roles and Activities; and (3) Neuro-QoL Stigma. The cognitive HRQoL domain includes two PROs: (1) Neuro-QoL Applied Cognition—Executive Function; and (2) Neuro-QoL Applied Cognition—General Concerns.

HDQLIFE End of Life Planning includes 16 questions on a 3- to 4-point Likert scale.^9,29^ This PRO measures the range of health behaviors, communication, and thoughts around ACP. Four subdomains exist (1) “Legal Planning,” (2) “Preferences for Care,” (3) “Death and Dying Preferences,” and (4) “Financial Planning.” Responses are framed around the participant's continuum for the ACP process, ranging from not thinking about a given behavior/communication/topic/action to engaging in said item. The “Legal Planning” subdomain (*n* = 3 questions) asks about advance directives, living wills, and the health care power of attorney.

“Preferences for Care” (*n* = 3 questions) discuss skilled nursing facility care and palliative and hospice care. “Death and Dying Preferences” (*n* = 5 questions) explore conversations about the dying/death process, location of death, and preferences about death, resuscitation (i.e., intubation/cardiopulmonary resuscitation), and funeral arrangements. “Financial Planning” (*n* = 4 questions) concerns one's insurance, estate, finances, and assistance with helping to make medical decisions if/when the capacity is lost. One question that is not part of any subdomain (but is computed within the total score) and to which a participant may opt out of answering (lack of applicability) concerns preparation for child care.

All PROs are scored using the T score metric (mean = 50; standard deviation = 10) according to the reference population under study (e.g., HD for the HDQLIFE measures and the general neurological or adult populations for the Neuro-QoL or PROMIS assessments, respectively). Higher T scores reflect more of the assessed domain (e.g., higher HDQLIFE End of Life Planning suggests more engagement with ACP activities; and a higher Neuro-QoL Anxiety reflects more anxiety).

### Statistical analysis

Demographic and clinical data (age, sex, ethnicity, education, CAG repeats) were described using means and standard deviations for continuous variables and frequencies and percentages for categorical variables. HDQLIFE End-of-Life Planning Total Scores were stratified using the baseline data into three separate groups: low (T score ≤40) versus medium (40 < T score <60) versus high levels of planning (T score ≥60). Comparisons were made using Kruskal–Wallis and Chi-square tests.

Linear mixed-effects models were used to assess the relationships between (1) baseline HRQoL/STB and ACP at follow-up and (2) baseline ACP and HRQoL/STB at follow-up. Random effects for participant were used to address the interdependence of the months 12 and 24 assessments within a participant. When assessing the if HRQoL/STB predicts ACP, 18 linear mixed-effects models (1 for each HRQoL/STB variable) were run with ACP as the outcome (assessed at months 12 and 24) with the following predictors: baseline ACP, an HRQoL/STB variable assessed at baseline (e.g., Chorea), as well as age, education, sex, stage, and baseline depression and anxiety. The same approach was used to assess the relationship between baseline ACP and symptoms at follow-up—this time with HRQoL/STB as the outcome.

We also performed an exploratory analysis because previous data suggested an association of advance directive completion with the sense of meaning and purpose, age, education, and HD stage.^4^ For this analysis, we used the multivariable model we developed that included physical, mental, social, and cognitive PROs/Assessments to test for an interaction between HDQLIFE Meaning and Purpose and each predictor variable. We also tested for an interaction between HDQLIFE Meaning and Purpose and each predictor variable for all linear-mixed effects models.

Two-sided α = 0.05 was used to assess for statistical significance. False-discovery rate-adjusted *p*-values accounted for multiple comparisons.^31^ Analyses were executed using SAS V9.4 (SAS Institute Inc., Cary, NC).

## Results

### Participant characteristics

Demographic and clinical differences existed across levels (low, medium, and high) of HDQLIFE End of Life Planning. Compared with the group with low engagement with the end-of-life planning, those with high engagement were, on average, 12.9 years older. On average, those with high engagement in planning had 2.4 more years of education than the low group. Higher physical symptom burden (i.e., HDQLIFE Swallowing difficulties and Neuro-QoL Upper Extremity Function) correlated with higher engagement with the end-of-life planning. No differences existed within ethnicity, race, marital status, or sex among the three groups (Table 1).

**Table 1.** Demographic Data by Advance Care Planning

Characteristic	Baseline advance care planning			Overall	***p***
	Low (≤40)	Medium (40–60)	High (≥60)		
Demographics	(*N* = 43)	(*N* = 227)	(*N* = 52)	(*N* = 322)	
Age (years)^a^	44.0 (15.46)	51.8 (11.95)	56.9 (10.54)	51.6 (12.72)	<0.0001
Female^b^	21 (49)	105 (46)	21 (40)	147 (46)	0.67
Hispanic of Latino^b^	1 (2)	5 (2)	3 (6)	9 (3)	0.48
Race^b^					0.17
African American	4 (9)	5 (2)	1 (2)	10 (3)	
Caucasian	37 (86)	218 (96)	50 (96)	305 (95)	
Other	2 (5)	3 (1)	1 (2)	6 (2)	
Unknown	0 (0)	1 (0)	0 (0)	1 (0)	
Education (years)^a^	13.2 (2.54)	14.8 (2.57)	15.6 (3.10)	14.7 (2.72)	0.0003
Marital status^b^					0.06
Single, never married	13 (30)	28 (12)	3 (6)	44 (14)	
Married	22 (51)	134 (59)	31 (60)	187 (58)	
Separated/divorced	6 (14)	53 (23)	14 (27)	73 (23)	
Living with partner	1 (2)	6 (3)	3 (6)	10 (3)	
Widowed	1 (2)	6 (3)	1 (2)	8 (2)	
CAG repeats^a^	45.4 (7.25)	42.8 (3.32)	43.2 (6.63)	43.3 (4.71)	0.06
HDQLIFE End of Life Planning^a^	34.8 (4.27)	50.0 (5.43)	65.4 (4.70)	50.5 (9.78)	<0.0001
Legal	35.6 (4.04)	50.6 (6.70)	60.4 (2.70)	50.2 (8.96)	<0.0001
Preferences for care	43.6 (5.40)	50.0 (6.97)	57.8 (8.54)	50.4 (8.05)	<0.0001
Death and dying preferences	39.7 (5.59)	49.6 (7.44)	61.5 (5.57)	50.2 (9.13)	<0.0001
Financial	41.2 (6.52)	48.9 (7.37)	57.1 (5.40)	49.2 (8.20)	<0.0001
Physical					
HDQLIFE Chorea^a^	50.5 (9.51)	52.9 (8.35)	54.3 (8.67)	52.8 (8.60)	0.17
HDQLIFE Speech Difficulties^a^	48.8 (8.97)	51.2 (7.88)	53.8 (8.36)	51.3 (8.19)	0.05
HDQLIFE Swallowing Difficulties^a^	48.8 (8.28)	52.0 (8.20)	55.6 (8.58)	52.1 (8.45)	0.0004
Neuro-QoL Upper Extremity Function^a^	43.8 (10.08)	41.1 (9.91)	36.8 (11.28)	40.8 (10.32)	0.0042
Neuro-QoL Lower Extremity Function^a^	46.8 (9.72)	45.3 (10.06)	42.7 (9.84)	45.1 (10.02)	0.18
Mental					
PROMIS Anger^a^	46.9 (12.46)	48.3 (12.49)	47.4 (12.11)	48.0 (12.40)	0.59
HDQLIFE Meaning and Purpose^a^	49.5 (9.08)	48.9 (9.69)	53.0 (7.42)	49.6 (9.38)	0.02
HDQLIFE Concern with Death and Dying^a^	48.4 (8.73)	51.0 (10.42)	48.7 (8.57)	50.3 (9.96)	0.17
PROMIS Anxiety^a^	52.1 (10.04)	53.8 (10.22)	53.2 (11.51)	53.5 (10.39)	0.45
PROMIS Depression^a^	51.0 (11.05)	51.1 (10.71)	50.6 (10.90)	51.0 (10.75)	0.90
Neuro-QoL Emotional^a^ and Behavioral Dyscontrol^a^	45.2 (9.07)	47.2 (11.15)	47.7 (10.90)	47.0 (10.86)	0.47
Neuro-QoL Positive Affect and Well-Being^a^	54.7 (7.81)	54.7 (8.68)	55.1 (8.48)	54.8 (8.51)	0.82
Problem Behavior Assessment—Suicide^a^	0.0 (0.00)	0.0 (0.22)	0.0 (0.14)	0.0 (0.19)	0.22
Social					
Neuro-QoL Ability to Participate in Social Roles and Activities^a^	46.8 (8.74)	46.3 (8.26)	45.7 (8.43)	46.3 (8.33)	0.67
Neuro-QoL Satisfaction with Social Roles and Activities^a^	50.3 (8.36)	46.9 (7.85)	47.3 (9.66)	47.4 (8.28)	0.05
Neuro-QoL Stigma^a^	49.0 (8.81)	51.4 (8.83)	52.5 (8.41)	51.3 (8.78)	0.14
Cognitive					
Neuro-QoL Applied Cognition—Executive Function^a^	39.8 (10.68)	36.4 (10.26)	33.0 (10.22)	36.3 (10.43)	0.01
Neuro-QoL Applied Cognition—General Concerns^a^	41.6 (7.68)	39.8 (9.58)	38.2 (8.67)	39.8 (9.24)	0.16

^a^ Mean (SD).

^b^ Frequency (%).

QoL, quality of life; SD, standard deviation.

### HRQoL and STB at baseline do not associate with HDQLIFE End of Life Planning in the future, and HDQLIFE End of Life Planning at baseline does not associate with changes to STB and HRQoL at follow-up

None of the HRQoL/STB measures at baseline predicted ACP at follow-up (Table 2). Similarly, after accounting for multiple comparisons, HDQLIFE End of Life Planning was not associated with 12- or 24-month reports of suicide or any of the physical, mental, social, and cognitive HRQoL PROs (Table 3). In addition, none of the models demonstrated a significant interaction with HDQLIFE Meaning and Purpose.

**Table 2.** Mixed Model Results of Baseline Health-Related Quality of Life Predicting HDQLIFE End of Life Planning at Follow-Up

Outcome	β [95% CI]	***p***
Physical		
HDQLIFE Chorea	0.09 [−0.09 to 0.26]	0.32
HDQLIFE Speech Difficulties	0.00 [−0.14 to 0.14]	0.99
HDQLIFE Swallowing Difficulties	−0.04 [−0.18 to 0.10]	0.59
Neuro-QoL Upper Extremity Function	−0.08 [−0.22 to 0.07]	0.30
Neuro-QoL Lower Extremity Function	−0.04 [−0.18 to 0.10]	0.55
Mental		
PROMIS Anger	−0.05 [−0.19 to 0.09]	0.51
HDQLIFE Meaning and Purpose	−0.02 [−0.14 to 0.09]	0.68
HDQLIFE Concern with Death and Dying	−0.01 [−0.14 to 0.13]	0.89
NQ/PROMIS Anxiety	−0.10 [−0.27 to 0.08]	0.28
NQ/PROMIS Depression	0.09 [−0.09 to 0.27]	0.31
Neuro-QoL Emotional and Behavioral Dyscontrol	0.03 [−0.13 to 0.18]	0.74
Neuro-QoL Positive Affect and Well-Being	0.03 [−0.13 to 0.19]	0.68
Problem Behavior Assessment—Suicide	−4.69 [−9.86 to 0.49]	0.07
Social		
Neuro-QoL Ability to Participate in Social Roles and Activities	−0.05 [−0.19 to 0.08]	0.44
Neuro-QoL Satisfaction with Social Roles and Activities	0.02 [−0.13 to 0.17]	0.80
Neuro-QoL Stigma	0.07 [−0.10 to 0.23]	0.43
Cognitive		
Neuro-QoL Applied Cognition—Executive Function	−0.02 [−0.16 to 0.11]	0.73
Neuro-QoL Applied Cognition—General Concerns	−0.08 [−0.21 to 0.06]	0.25

Adjusted for baseline levels of HDQLIFE End of Life Planning and age, education, sex, stage, depression, and anxiety.

CI, confidence interval.

**Table 3.** Mixed Model Results of Baseline HDQLIFE End of Life Planning Predicting Symptoms at Follow-Up

Outcome	β [95% CI]	***p***	***q***
Physical			
HDQLIFE Chorea	0.04 [−0.04 to 0.12]	0.35	0.75
HDQLIFE Speech Difficulties	0.08 [−0.01 to 0.18]	0.07	0.25
HDQLIFE Swallowing Difficulties	0.04 [−0.06 to 0.14]	0.43	0.75
Neuro-QoL Upper Extremity Function	−0.03 [−0.13 to 0.07]	0.56	0.75
Neuro-QoL Lower Extremity Function	−0.03 [−0.13 to 0.07]	0.58	0.75
Mental			
PROMIS Anger	0.18 [0.03 to 0.32]	0.02^a^	0.11^a^
HDQLIFE Meaning and Purpose	0.04 [−0.08 to 0.15]	0.51	0.75
HDQLIFE Concern with Death and Dying	0.02 [−0.10 to 0.13]	0.79	0.86
NQ/PROMIS Anxiety	0.01 [−0.13 to 0.14]	0.94	0.94
NQ/PROMIS Depression	0.13 [−0.00 to 0.25]	0.05	0.23
Neuro-QoL Emotional and Behavioral Dyscontrol	0.11 [−0.02 to 0.24]	0.08	0.25
Neuro-QoL Positive Affect and Well-Being	0.01 [−0.10 to 0.11]	0.87	0.92
Problem Behavior Assessment—Suicide	0.00 [−0.00 to 0.01]	0.20	0.51
Social			
Neuro-QoL Ability to Participate in Social Roles and Activities	−0.03 [−0.14 to 0.09]	0.66	0.79
Neuro-QoL Satisfaction with Social Roles and Activities	−0.13 [−0.23 to −0.02]	0.02^a^	0.11^a^
Neuro-QoL Stigma	0.14 [0.03 to 0.25]	0.01^a^	0.11^a^
Cognitive			
Neuro-QoL Applied Cognition—Executive Function	−0.05 [−0.17 to 0.08]	0.46	0.75
Neuro-QoL Applied Cognition—General Concerns	−0.05 [−0.16 to 0.06]	0.39	0.75

Adjusted for baseline levels of outcome and age, education, sex, stage, depression, and anxiety.

^a^ Not significant after false-discovery rate adjustments.

## Discussion

Before our study, no information was available on whether ACP relates to changes in HRQoL over a 12- and 24-month time frame in people with HD. This study contributes two key findings that are important for palliative neurology. First, STB and HRQoL PROs do not significantly contribute to patient engagement with ACP. Second, participating in ACP discussions does not cause untoward effects as we observed no changes to STB and physical, mental, social, and cognitive HRQoL in HD over the subsequent 12 and 24 months.

Overall, navigating ACP discussion is an essential role for clinicians caring for patients with HD. Our study shows that these discussions do not worsen STB or HRQoL and further research on ACP is warranted to see how ACP may lead to improved care in patients with fatal neurodegenerative disease like HD.

*A priori*, we had hypothesized that HRQoL PROs would contribute modestly to ACP. However, our findings suggest otherwise. Other factors may more substantially contribute to ACP engagement in HD. Previous studies in other serious illnesses suggest coping behaviors,^32^ personalities, self-esteem, illness-specific factors (e.g., stage, age), care partner involvement, behavioral change knowledge, prognostic awareness, readiness to talk about the future, and the attitudes of health care professionals.^13^

Our study is not without its limitations. We analyzed data retrospectively from a longitudinal multi-center observational study dedicated to creating PROs responsive to the HRQoL needs of people with the HD mutation. However, additional insights to understand our findings are notably lacking. Our approach unfortunately does not provide qualitative insights into understanding ACP at each visit and its interpretation with HRQoL for each person with HD and their loved ones.

Indeed, we would foresee that a mixed-methods approach would allow for a greater understanding of our quantitative findings. Another limitation of our findings includes that our dataset did not record who initiated ACP. That is, uncertainty surrounds as to who initiated the process (i.e., the patient, care partner, health care professional, or another third party) or the reasons behind engaging in various forms of ACP (i.e., poor health from other non-HD causes, progression of HD, recent hospitalizations, or falls).^33^

Therefore, we recommend that future work within this area should use mixed methods to explore the framing of ACP discussions in HD, their essential components, and barriers to the occurrence.

Reassuringly, and in agreement with prior research, we found that preparing for the end of life does not relate to increases in STB or decreases in HRQoL at 12 and 24 months. While we did observe marginal worsening in mental and social HRQoL at follow-up, specifically in anger, depression, satisfaction with social activities, and stigma, these did not reach significance after correction for multiple comparisons.^30^ Nevertheless, this warrants further study in the ACP context.

Interestingly, depression and anger comprise stages of grief, per the “stage theory of grief” model.^34^ We hypothesize that people with the HD mutation who engage in ACP may concurrently engage in “grief work” while experiencing the loss of independence and respect (hence the trend toward worsening stigma) and thus progressing through these different grief stages (e.g., depression and anger). Corroborating such a hypothesis in the future would perhaps recapitulate a recently published longitudinal observation among people with advanced cancer: those who completed a living will or resuscitation preference predicted negative mental HRQoL at two months—specifically increased grief.^35^

Future work should clarify if an association exists between ACP engagement and measurements of grief within HD, and assess for markers of grief within an HD care partner while incorporating the care partner's HRQoL. If psychosocial distress appears during the ACP process in this population, then the consideration of integrating ACP within an adapted psychotherapeutic neuropalliative intervention might also be warranted.^36^

Our data suggest that STB and HRQoL are not significantly related to ACP practices within the HD population. Thus, our data should comfort stakeholders that engagement in ACP does not provoke emergent STB, especially within a disease where STB's lifetime prevalence nears 20% to 30% and suicide leads as a cause of death.^11^ Our findings suggest the importance of future investigation into the non-significant worsening signals observed in mental and social HRQoL, especially in stigma, depression, anger, and satisfaction with social roles and activities.

We conjecture that an unmeasured personality or psychiatric (co-morbid) factor may contribute to residual confounding—and one's predisposition to engage in ACP in this population, especially since our observational study neither promoted nor eschewed ACP. In the interim, our study can help tailor HD-specific neuropalliative interventions that meet the ACP needs of this unique and complex patient population.

## Abbreviations Used

ACP: advance care planning
APDA: American Parkinson Disease Association
BMS: Bristol Myers Squibb
CI: confidence interval
DCL: diagnostic confidence level
HD: Huntington disease
HDSA: Huntington's disease Society of America
HRQoL: health-related quality of life
HSG: Huntington Study Group
NCATS: National Center for Advancing Translational Sciences
NIA: National Institute of Aging
NIH: National Institutes of Health
NINDS: National Institute of Neurological Disorders and Stroke
PBA-s: short Problem Behavior Assessment
PROs: patient-reported outcomes
QoL: quality of life
SD: standard deviation
SFs: short forms
STB: suicidal thoughts and behavior
TFC: Total Functional Capacity
UHDRS: Unified Huntington Disease Rating Scale
